# Quasi-Qualitative Evaluation of Progressive Counting in Secure Accommodation in Scotland: an Exploratory Cluster Case Study

**DOI:** 10.1007/s40653-017-0184-6

**Published:** 2017-08-07

**Authors:** Ian Barron, Jane Kim Tracey

**Affiliations:** 1 Old Medical School, School of Education and Social Work, University of Dundee, Rm 0.1.13, Dundee, Scotland, DD1 4HN UK; 2 St Mary’s Kenmure, Bishopbriggs, UK

**Keywords:** Brief exposure, Therapy, Secure care, Juvenile detention

## Abstract

This was the first study to introduce a brief exposure therapy, within a trauma-informed phase approach, into a secure facility in Scotland. An exploratory cluster case study was used to identify the perceptions of the first three youth who completed Progressive Counting (PC), a novel approach to brief exposure, within the Fairy Tale Model. The youth and their newly trained therapist received a semi-structured interview at 3 months following the completion of therapy. In-depth interviews involving rating scales and open-ended questions were conducted by telephone and digitally recorded. A quasi-qualitative approach was used to analyze data. Independent ratings by two researchers checked for inter-rater reliability. A retrospective expert rating was provided for treatment fidelity. Youth reported a range of gains in relation to program objectives including reduced distress and putting trauma into the past. Challenges of implementation are discussed. More rigorous evaluation of PC, including randomized control trials, is needed before PC can be recommended as a treatment of choice.

Children in secure accommodation in Scotland, as elsewhere in the world, have been exposed to a wide range of adverse childhood experiences (Abram et al. 2007). Sudden traumatic loss, domestic violence, emotional, physical and sexual abuse, neglect, and gang violence are some of the traumatic events experienced (Baglivio et al. 2014). In the only published Scottish study to explore trauma exposure for youth in secure accommodation, Barron and Mitchell (2017) found all seventeen youth in one facility reported multiple cumulative traumatic events. Exposure included murder, suicide, siblings taken into care, parental imprisonment, assaults, gang violence, drug overdoses, domestic violence, and rape. Similarly, Kibble Education and Care Centre (2011) in an unpublished Scottish study, identified 13 different categories of abuse experienced by youth. International studies suggest trauma exposure for incarcerated youth is far higher than in the general child population, ranging from 25 to 90% respectively (Arroyo 2001; Costello et al. 2003). Prevalence figures in both general child population and incarcerated youth studies vary widely because of the differing definitions and methodologies used.

The consequences of cumulative traumatic events, especially if abuse is by the caregiver, include poor physical and mental health, troubled relationships, underachievement, behavioral difficulties, criminality, and problems with employability (Van der Kolk 2005). As with exposure, resultant posttraumatic stress, suicide, and other mental health symptoms are significantly higher in youth in detention than in the general child population (Bhatta et al. 2014; Stimmel et al. 2014). Specifically in Scotland, 65% of youth in one secure facility were found to meet the criteria for PTSD and depression and 18% met the criteria for dissociation (Barron and Mitchell 2017). In comparison, around 30% of juveniles in detention in the United States present with PTSD (Kerig and Becker 2012). Again, prevalence statistics vary widely from 2 to 52% (Kerig and Becker 2012; Kerig et al. 2014). Despite these concerning figures, youth in Scotland are not placed in secure facilities because of mental health concerns, rather they are placed because of behavioral difficulties where they put themselves and others at risk. These facilities are similar to juvenile detention, however, in Scotland they are located within residential care, rather than the juvenile justice system (Scottish Executive 2006). Secure facilities in Scotland seek to stabilize behavior and promote social, emotional, and behavioral change (Barron and Mitchell 2017). Despite containment and behavior change programs, youth have high recidivism rates and poor behavioral, educational, and employment outcomes (Trulson et al. 2005). Mears et al. (2011) argue that these poor outcomes are due to the lack of empirically supported programs as well as the failure to understand that trauma is the driving force influencing behavior. Without addressing youth trauma history, any behavioral change is likely to be of short duration (Abram et al. 2015). Attempts at addressing youth trauma within secure facilities are in their infancy (Marrow et al. 2012). The first study in Scotland to implement and evaluate a trauma-specific program in a secure facility found a significant reduction in youth subjective disturbance (Barron et al. 2017). Youth had received the group-based Teaching Recovery Techniques program based on cognitive behavioral theory. The randomized control trial, however, failed to find a significant difference between intervention and control groups on standardized measures of posttraumatic stress, depression and dissociation. The small (*n* = 17) and heterogeneous sample suggests caution in interpreting the results. The authors concluded that individual psychotherapy may better address the issue of youth apprehension in sharing traumatic experiences with youth they were living with. The current study is the first to introduce an individualized brief exposure therapy within a trauma-informed phase approach to a secure facility in Scotland. Progressive Counting (PC) is a recently developed variant of the counting method (Greenwald, 2008a, b; Greenwald 2013). PC aims to reduce posttraumatic stress and other trauma related symptoms. PC involves the therapist counting out loud in increasing amounts, while the client imagines the traumatic event in between a good beginning image and a good end image. PC has been found to be about as effective as EMDR (Greenwald et al. 2013; Greenwald et al. 2015) and less emotionally dysregulating for reactive youth (Greenwald et al. 2015). Recent case studies with youth have found that two-day intensive PC reduced a traumatized female’s distress sufficiently to disclose abuse and receive child protective services (Greenwald 2014). In a cluster case study, an 11 year old girl who experienced family bereavement and repeated molestation, and a twelve year old girl who experienced loss and a physical injury, both reported reduced posttraumatic stress symptoms to non-clinical levels. Treatment was of short duration and lasted 12 and four weeks, respectively (Greenwald, 2008a, b). In an archival adult study of the first three cases with one therapist, PC was found to reduce PTSD with progress maintained at one year follow-up (Jarecki and Greenwald 2015). PC’s impact has also been explored in international settings on a group basis. Workshop participants (*n* = 232) in over six countries, who experienced only 5 min of PC for a minor upsetting memory, reported reduced disturbance. Follow-up of 128 participants, who experienced a second individual PC session, supported these results (Greenwald and Schmitt 2010). PC studies, however, are in their infancy and most have been conducted with therapists in training, workshop participants and volunteers from the community (Greenwald 2012; Greenwald et al. 2013; Greenwald et al. 2015). The generalization of these results to clients seeking therapy is therefore limited. Further, nearly all PC studies have been conducted with the author of PC as the principal investigator. This leaves studies open to the challenge of researcher bias. Potential mechanisms underlying PC have begun to be explored. In a study involving mental health professionals (*n* = 109) who experienced a single session of PC, seven potential mechanisms were identified, i.e., emotional processing, desensitization, meaning-making, dual focus, distraction, and distancing as well as the quality of therapist-client interactions (Greenwald 2012). Follow-up of twenty-six therapists, who experienced an individual session for a more distressing memory and who commented at each round of imaginal exposure, provided further support for these mechanisms. The impact of PC has been also explained in terms of facilitating memory consolidation (Lasser and Greenwald 2015). In short, memory consolidation involves three key phases: (i) activation of the target memory and associated lesson learned (often a negative core schema), (ii) destabilization of the target learning with a dis-confirmatory experience, and (iii) modifying the target learning by guiding more dis-confirmatory experiences within a five-hour period (Ecker et al. 2012).

The current study builds on previous adolescent and adult PC case studies and research. It explores the novel delivery of a brief exposure therapy (PC) within a trauma-informed phase model of treatment (Greenwald 2009) in a secure facility in Scotland. The study’s main aim is to generate discussion of the benefits and challenges of PC for this population. A cluster case study design was used to identify the experience and perceived impact of PC. The first three youth who completed PC and their newly trained therapist were interviewed following treatment.

## Methods

### Research Design

As this was a novel intervention applied in a new context, an exploratory cluster case study design was used to examine the perceived experiences and outcomes of the first three youth in a secure facility who completed PC, and their newly trained therapist. A quasi-qualitative approach was used to analyze data from semi-structured interviews conducted at three month follow-up. Codes and themes were assessed for inter-rater reliability. An expert retrospective rating was sought on videoed sessions for therapist protocol adherence. University Research Ethics Committee approval required active informed consent by (i) the facility manager, the youth’s temporary legal guardian with authority to grant consent on the youths’ behalf; (ii) the therapist and (iii) active informed assent was required by the three youth, who agreed to their cases being reported.

### Participants

The facility is located in a large Scottish city. Youth were placed from all over Scotland. The maximum number in the facility was twenty-one (12–18 year olds). The three youth were purposefully selected because they were the first to complete PC. Participants received PC from a newly trained therapist. The therapist, a secure interventions practitioner in her 40s, had worked in secure settings for six years. She had no previous experience of trauma-specific programs or brief exposure therapy. Two youth were aged 15 years and one 17 years. All were Scottish Caucasian males and placed in secure because they posed a risk to themselves and others in the community. For all three, this included gang violence, substance misuse, self-harm, and attempted suicides. From trauma history interviews, all had experienced multiple and cumulative trauma from domestic violence, physical and emotional abuse, physical assault, numerous sudden traumatic loss, and neglect. All families were living in relative poverty. Youth had been placed in secure for 24, six, and 25 months, and had experienced five sessions of PC, four sessions, and five sessions, respectively. One target was processed per session. At the time of interview, one was in the community, one in a residential facility, and one in the same secure facility. The therapist had seen five youth previously, none of whom had reached PC. All had failed to buy into the earlier phases of treatment and had dropped out. This was mostly related to the therapist learning to applying new treatment skills. Youth had not been persuaded that they had been traumatized and needed treatment. These youth were not included in the study.

### Treatment and Training

PC was developed as a trauma resolution therapy that aims to reduce symptoms of posttraumatic stress, anxiety, anger, guilt and depression, and increase self-competence and confidence. In brief, PC involves the client imagining "a ‘movie’ of the entire story of the trauma memory from beginning to end, while the therapist counts out loud from 1 to 10, the next time to 20, the next time to 30, etc., until no further memory-associated distress remains. As is standard across trauma resolution methods, all the memories and memory details are generated by the client” (Greenwald 2014, p. 329). A minor upsetting memory is used as a test run first. Repeated imaging of the movie, where the traumatic memory is in between a good beginning image and good ending image, while listening to the therapist count, appears to facilitate the healing of the traumatic memory.

PC was the final phase of the Fairy Tale Model (FTM), a trauma-informed phase model of treatment. FTM is described as a generic or trans-diagnostic treatment approach that allows clinicians to address the wide range of problems presented by youth in secure facilities (Greenwald 2013). FTM includes: assessment of strengths, resources, trauma/loss history, life situation, and presenting problems, identification and enhancement of goals and motivation, trauma-informed case formulation and treatment contracting, stabilization, consequential thinking, avoiding high risks, coping and affect tolerance skills, resolution of trauma and loss memories (PC), consolidation of gains, and anticipation of future challenges. The therapist, with 11 others, received three days training in FTM and one day in PC, by Dr. Greenwald. The cohort received monthly group supervision sessions. Seven half-day sessions were held via video conferencing and four days were face-to-face. Supervision involved therapists showing videoed therapy sessions and receiving expert feedback on protocol adherence, understanding trauma reactions, and responding to youth.

### Interviews

Interviews with the therapist and youth were held three months following completion of therapy. Telephone interviews were conducted by the researcher. Youth were provided with a phone in their room with no one present. Interviews were piloted with one youth and a therapist not involved in the study. Following the pilot, two questions were added to the youth interview, i.e. what advice would you give secure facilities for PC and what is the best way to motivate youth to engage in therapy? Questions included rating scales and open ended questions to enable responses to be quantified, and themed for meaning. Interviews were digitally recorded, transcribed verbatim by an independent company, and checked for accuracy by the researcher. Interviews lasted 45–50 min for the therapist and 20–25 min for youth.

The youth interview was based on the phases of treatment and included the following questions: What youth liked and disliked about PC and scaling the following from 0 to 10, where 0 was no change and 10 was the most change they could imagine, impact of PC on their lives, awareness of traumatic events, motivation to heal and change, imagining a better future and steps to achieve this, avoiding high risk situations, making better choices and knowing choices have consequences, reduced stress, resolved traumatic memories, and anticipating risky situations. Feelings before, during, and after sessions were sought, and if there were negative consequences associated with PC. The therapist interview mirrored youth questions to enable triangulation of data as well as identify therapist specific issues. Questions included: youth understanding of trauma and rating-scales of intervention effectiveness on (i) assessment, (ii) stabilization; (iii) case formulation; (iv) avoiding high risks; (v) resolution of trauma; (vi) consolidation of gains; (vii) anticipation of future challenges; (viii) report writing and meetings, (ix) and benefits, and challenges for youth, therapist, and the secure facility.

### Analysis

Initial analysis of interview data was completed by the researcher, a reader in trauma studies and an accredited Eye Movement Desensitization Reprocessing (EMDR) practitioner. A quasi-qualitative approach using a six-step systematic thematic analysis was used (Braun & Clarke 2006). The term quasi-qualitative is used because of the counting and rank ordering of codes, and statements as well as the identification of themes. The procedure is as follows: 1. Familiarization through re-reading data and noting initial ideas for patterns of meaning; 2. Codes were systematically generated from data with statements of meaning collated under each code. Codes were named, where possible, using participants words; 3. Codes were collated with the set of statements into identified themes; 4. Codes and statements were counted, and rank ordered, and themes were reviewed and checked against statements; 5. Themes were finalized; 6. Report writing enabled a further analysis with exemplifying quotes for codes and themes. The three adolescents’ quotes are identified by the letters A, B, and C. Codes names are reported with the number of statements, and codes per theme are totaled. Therapist interview responses are presented as quotes and themes to provide a contextual narrative to compare with the quasi-qualitative analysis of youth interviews. Statements, codes, and themes were analyzed across the three youth, and therapist to explore commonality, and difference in perceptions. Therapist and youth responses were compared for the benefits, and challenges of PC. Inter-rater reliability involved a postgraduate research assistant, independently reviewing the names of the codes and themes. Treatment fidelity analysis involved requesting Dr. Greenwald to retrospectively rate (Two months following previous supervision sessions) from 0 to 10 (where 0 was no adherence and 10 was complete adherence) the therapist’s protocol adherence in videoed therapy sessions brought to supervision.

## Results

### Youth Interview

Inter-rater analysis led to changes in the name of five themes. Changes reduced wordiness rather than conceptual difference. Treatment fidelity was scaled as 6–7, a rating considered representative of a novice trauma therapist. This fit well with the purpose of the study which was to assess a novice therapist’s use of PC. In practical terms, this meant “adequate on the essentials, but with plenty of errors.” This was “good enough” for a novice therapist but is not indicative of an experienced therapist’s work. Six codes from 27 statements referred to youth *liking PC including having the choice to disclose and the reductions in disturbance*. Codes with the number of associated statements in brackets were: PC works (*n* = 8); learning how to deal with situations (*n* = 5); privacy as a choice (*n* = 4); the worker’s qualities (*n* = 3); realizing acting-out is not worth it (*n* = 2); and it is easy to do (*n* = 1). No dislikes were reported, however, 4 statements referred to feeling emotional during PC. Representative quotes are “I’m using the strategies I learned in day to day life. When you have to think of a situation and go over it in 10, 20, 30 seconds, I found that important because it’s like thinking of what happened in 10 seconds, but then what else can happen in 20 seconds. So it’s thinking about the whole picture, so I went, ‘Do you know what, it doesn’t matter. It isn’t even worth it’ (A)**.** “It was good because X (therapist) was there. It’s easy to do, lying back and counting the numbers. It really got rid of your biggest fears. It was very emotional. It depends what emotion you were doing. You try and re-enact the emotions and the different situations. You get used to the emotions” (B), and “I didn’t have to speak about anything I didn’t want to. Any other program I’ve done, they try and get you to do stuff that you don’t want to.” (C).

### Impact

On a scale from 0 to 10, where 0 is ‘no effect’ and 10 is ‘most effective,’ impact was rated from 6 to 10 with an average of **8.7**. Fourteen codes were identified from 29 statements indicating *improved emotional and behavioral functioning as well as positive identity change*. Codes were: I think about the consequences and stop (*n* = 7); it helped (*n* = 3); control emotions now (*n* = 3); processed trauma and doubts (*n* = 2); stopped criminal behavior (*n* = 2); avoiding risks (*n* = 2); a better perspective (*n* = 1); recognize maturing now (*n* = 1); a positive identity (*n* = 1); improvements in real life (*n* = 1); others have noticed change (*n* = 1); it’s important to do the whole program (*n* = 1); and it may not help some (*n* = 1). The worker and experience were highly valued (*n* = 3).

“Definitely a 10, it may not help some but it definitely helped me. I’ve been out there and kept my head down. It opens your eyes. Nowadays I think about it before, and then I’m like, ‘No, that’s not worth it,’ so I won’t do it” (A). “Ten definitely. I’m more mature, healthy, wise young man. X does a great job. It was just everything, that’s like gold dust. I matured from it and it put a lot of my doubts at the back of my head away, and got rid of them” (B), and “Six because it’s not only me that’s noticed the change. Other people have noticed it. I’d just react. It’s stopped me from stealing cars, doing crime, because I’ve stopped to think about things, when I’m sober anyway. It helped me control my emotions. Like see when I’m getting angry I used to just explode. Now I can bring myself down to 0” (C).

### Awareness of traumatic events

On a scale from 0 to 10, where 0 is ‘no awareness’ and 10 is ‘fully aware,’ youth rated from 7.5 to 10, with an average rating of **8.5**. Seven codes were identified from 10 statements indicating *a shift from re-experiencing trauma to remembering events*. Codes were: the difference between remembering compared to re-experiencing (*n* = 3); reflecting on past traumatic events (*n* = 2); events can happen (*n* = 1); see the extent of events now (*n* = 1); recognizing the negative impact on self (*n* = 1); gaining new understandings (*n* = 1); and able to speak about the trauma (*n* = 1).

**“**Ten. It’s made me think about how stuff could happen to me. It was just obviously going through the crime (PC), that’s a shock to the system” (A). “Eight, I’m still 100% aware of them. It’s just they’re not much of a problem to me anymore. I can understand them and just get on a bit more” (B), and “Seven and a half. I’m aware of the traumatic events that have happened in my life and the result. I’m always up and I speak of it” (C).

### Motivation to heal

On a scale from 0 to 10, where 0 is ‘no motivation’ and 10 is ‘highly motivated,’ youth rated from 5 to 10, with an average rating of **8.3.** Seven codes were identified from 17 statements indicating *increased motivation and sense of purpose*. Codes were: thinking rather than triggered response (*n* = 5); recognizing goals and obstacles to address (*n* = 4); tangible feeling of being motivated and sense of purpose (*n* = 3); got better (*n* = 2); sense of purpose seen as a reason for making best use of program (*n* = 1); sense of own immaturity (*n* = 1); and still desire to engage in some risky behavior (*n* = 1). “Definitely a 10. I have really got better. I feel motivated. I feel like I can think stuff through more now than just fight or flight. I keep thinking of stuff more than just going out and doing it, and I don’t regret it after” (A). “Ten. I was committed to the program 100%. I wanted to finish it and get it done. Cause if I’m wanting to move on with my life. I’m going to have to get things done, out the way, things answered” (B), and “About a 5. I don’t really think about the future? I still think about the situations that I’m in but still up for getting into a bit of bother?” (C).

### Imagining a better Future

On a scale from 0 to 10, where 0 is ‘unable to imagine a better future’ and 10 is ‘a good image of the future,’ youth rated from 5 to 8, with an average rating of **6.7.** Seven codes were identified from 17 statements indicating *an improved commitment to adolescent self-identified goals*. Codes were: discovering a future goal through a past good relationship (*n* = 5); able to put behavior into perspective (*n* = 4); real life goals to look forward (*n* = 3); gained a permanent ‘brighter’ picture of the future (*n* = 2); holding onto and using good and bad endings (*n* = 1); seeing self as on a journey towards to the good ending (*n* = 1); and immediate access to the good ending (*n* = 1). “Eight, because it’s always about a brighter picture. There’s always a better future. Say, I did react over something. I’d have something else going for me*.* I look forward to the future. I’m starting college in August so that’s a positive straight away.” (A). “Seven, because so far I’m following the road of the good ending. Having a bar-b-que in the back garden. All my pals and everybody I know, they’re all having a good time” (B), and “About a five because I’ve been in a long-term relationship and I didn’t really think of it until X opened my eyes to it. That I could have a future and I’ve been with her for a few years now” (C).

### Avoiding High Risk Situations

On a 0–10 scale, where 0 is ‘no avoidance of high risk situations’ and 10 is ‘completely avoiding high risk situations,’ youth rated 4 to 10, with an average rating of 8**.** Five codes were identified from 10 statements indicating *increased awareness of risky situations and the identification of strategies to avoid risks.* Codes were: able to make a past-present comparison of behavior change (*n* = 3); aware of putting self at risk occasionally but not liking it now (*n* = 3); avoiding high risk made a difference (*n* = 2); developed a plan to keep on track (*n* = 1); and awareness of negative consequences as a motivator (*n* = 1). “Ten, because I used to not be good if somebody spoke to my girlfriend or someone in my face but if somebody does say something, I just shake it off, like “Oh, it doesn’t matter.” (A). “Six. I don’t get myself involved in any shenanigans now. Sometimes stuff just happens and I feel the need to do whatever I’m doing. I’ve got a plan in place to keep me on track and make sure I’m outa bother and consequences for the wrong actions” (B), and “It helps me when I’m clear minded. I can think about it. An 8” (C).

### Making Better Choices

On a 0–10 scale, where 0 is ‘not making better choices at all’ and 10 is ‘always making better choices.’ youth rated from 6 to 10 with an average of **7.7.** Seven codes were identified from 11 statements indicating the *adolescents reported increased responsibility for making better choices.* Codes were: using goal of reconnecting with family (*n* = 2); aware and taking responsibility for problem behavior (*n* = 2); better sense of identity and maturing (*n* = 2); noticing making better choices (*n* = 1); uses idea of ‘back on track’ (*n* = 1); strategy to use (*n* = 1); thinking before acting (*n* = 1); and recognizes PC contribution to success (*n* = 1). “Ten. Overreacting to something that’s my own fault, nobody else’s. Just counting to ten” (A). “Seven. I’m thinking about things before I do them. Taking responsibility after my actions, good or bad. I think I’m just a better person and more mature” (B), and “About a 6. I have started to make better choices, have started to get back on track with my family. We didn’t speak at all. I cut myself off and now I’ve got a good relationship with my granny and my sister” (C).

### Reduced Stress

On a 0–10 scale, where 0 is no reduction in stress and 10 is not feeling stressed, youth rated 6 to 10, with an average of 8.7. Eight codes were identified from 14 statements indicating *less stress and a greater openness to share experiences*. Codes were: remembering PC thinking and breathing (*n* = 4); triggers stressful memories (*n* = 3); 100% better (*n* = 2); attribute success to PC (*n* = 1); feel relief (*n* = 1); speak about trauma now (*n* = 1); valued being asked (*n* = 1); and valued speaking within and beyond sessions (*n* = 1). “Ten. What X has done with counting. She started with 10 and then she’d say just think, and make you go again but make you go to 20 and it releases the stress. So you’re taking that deep breath, where she is saying that, and I’d be like thinking” (A). “It brings back stressful memories, but obviously after it, when you get them out the way, the trauma and all that, it gets rid of them, and it makes them 100% better” (B), and “Probably a 6. I didn’t really speak about traumatic things in my life because I didn’t feel I needed and X spoke to me, and I think I could speak to X after PC” (C). Table 1 highlights the positive shift in feelings before during and after therapy.

**Table 1:** A, B and C’s feelings before, during, and after PC

Before	During	After
I never thought about things as much until I started. I thought a bit but I was a different person from the person I am (A). I tried my hardest not to think about it but I did. Angry, stressed, and unwell (B). Traumatized, upset and nervous. I didn’t know what was going to happen. Who cares attitude (C)?	I felt a lot better. I could open up. It was like I was being mended (A). Helped. I liked it because it was easy. It was with someone I got on with. Very comfortable (B). It’s okay if you don’t want to talk. Feel special, I started to realize… (C)	I felt better. Got all info I needed. Cured. I felt absolutely amazing (A). Proud to complete and get the things out of the way (B). Not as much weight on my shoulders (C).

### Reducing bad Memories

On a 0–10 scale where 0 is no reduction in traumatic memories and 10 is complete reduction in traumatic memories, youth rated 5 to 8, with an average of **7.7.** Nine codes were identified from 20 statements indicating t*he processing of trauma memories,* i.e. *putting the past into the past but not forgetting.* Codes were: peace and quiet of PC (*n* = 4); still hold the memory (*n* = 4); goal to get on with life/be oneself (*n* = 3); know how to deal with past (*n* = 3); puts the past into the past (*n* = 2); expectation of calm (*n* = 1); getting over trauma (*n* = 1); feel for the victim (*n* = 1); and competence in processing trauma (*n* = 1). “Ten, because by the end you should be calm. You say it in your head, my crime, that’s a hard one, because I feel for the person but I’ve got to get on with my life” (A). **“**Eight. I can move on with my life and get rid of the bad memories, and carry on being myself” (B), and “About a 5. It helped me bury the hatchet of some bad memories, but I’ve got some there. I like the peace and quiet. You don’t really get that anywhere in this building” (C).

### Anticipating Future Challenges

On a 0–10 scale, where 0 is ‘no anticipation’ and 10 is the ‘highest anticipation of future challenges’, youth rated 6 to 10 with an average of **7.7**. Five codes were identified from 8 statements indicating *an improved capacity to anticipate risky situations*. Codes were: using goals as a motivator (*n* = 5); committed to not wanting challenging situation (*n* = 1); acknowledging feelings and risk (*n* = 1); assessing risky situations with good and bad endings and deciding ‘it’s not worth it’ (*n* = 1); and choosing avoiding strategy (*n* = 1). “Ten. I don’t want another risky situation. Just think of my child, my girlfriend, think of how I lost them in the first place” (A). “Seven. Sussing out (assessing) the situation, looking at different ways it could go and then realizing if it’s worth it for me. Being aware, to make the best situation out of a bad one” (B), and “Six. I now know what days it’s alright to go to my pals. I know if it’s going to be quiet a drink and what days it’s going to be fighting. I used to go every day but now I wait back” (C).

### Advice to Help Other Youth Engage

The three youths provided four main suggestions to help other youth engage. These were: *Information from youth to youth:* **“**Show them our comments about the program, to show them that we did do it;” *Clear explanation:* “This is what’s in the program and this is what you’re going to be doing;” *Ensure commitment:* “I think it is good and for it to work, kids have to be committed because there’s a lot of stuff you don’t know about the counting;” and *Provide good relationships:* “You have to have a good bond with the person to trust them, patience. Her enthusiasm to try and make you better, and get things out of the way so you can have a better life. She cares a lot.”

### Therapist Interview

#### Benefits for Youth

The therapist’s comments, indicated *PC was a positive experience leading to increased motivation, observable change, and future possibility.* “Young people got a lot out of it, working towards their future goals, and seeing that you’d overcome difficulties, and able to achieve things. It really made a change in the young person, which was something care staff commonly said. They could see the young person was putting a lot of effort into it, getting something from it, discussing what they were doing, and how they benefitted. Maybe the youth were more open than they had been before. It was difficult in terms of youth wanting to work through it a bit quicker, a bit more intensive. The way things were timetabled, they didn’t always have that opportunity.”

#### Benefit for the Therapist

The identified theme was PC provided *a new way of understanding and increased confidence in addressing youth trauma.* The therapist commented “I liked PC because as long as the young person was up for it, some of them benefitted from doing it in longer sessions. I liked that flexibility." In addition, "it brought staff back to discussions about trauma. It’s a nicer way of working compared to a risk model where you are talking about behaviors this person has to stop. Something about the language, rather than it being about ‘this person is bad, they must change.’ You start to think about what the youth have been through, why is this behavior there, let’s do something about it. Something about the environment you create with it.”

In addition, PC “provided a structure that wasn’t too rigid. Even though it was scripted, it was led by the young person. That is where you got your flexibility. Initially, it provided a lot of confidence in working with someone on their trauma. Trauma has always been something I would like to do something about it, but not sure what to choose (intervention). With this, I felt very confident that we (other PC trained therapists) could work with young people and it would be successful”.

#### Trauma History and Case Formulation

The identified theme was *the value of PC bringing trauma-informed assessment, understanding and a method of case formulation.* “Definitely think more about highlighting the impact of trauma in assessment, and in presenting problems. I think about that in a different way, in terms of trauma-informed care." For example, "Impulsivity and managing emotions, thinking about trauma in relation to those things. Treatment contracting is much different ‘cause we generally were taught, what’s the behavioral difficulty, and how are we gonna look at it? We now tag on 'can we resolve the trauma'? Before we would have just left the trauma.”

#### Stabilization and Avoiding High Risk

The theme from the therapist’s comments was *PC enabled the thinking of alternatives for the young people along with observable behavior change*. “Reports from family and unit staff that they’ve done something with PC. Put PC into practice. Sometimes a young person will tell them, ‘This is what I’m doing and this is why I’m doing it, because this is what I’ve been taught.’ You see them being able to identify the problem and then work through the steps. It helps the youth think about alternatives.”

#### Resolution of Trauma and Anticipation of Future Challenges

The identified theme was *PC led to reduced distress, improved thinking and helped youth consider their future needs.* “PC helped minimize the rawness of feelings. It made them think about what else was out there for them, what could possibly happen, like good things and bad things, and what could I do in that situation? The framework sheets showed the treatment aims and where the gaps still are for the future.”

#### Trauma within Report Writing and Meetings

*Therapist increased knowledge, skill and confidence in trauma-informed communication was the identified theme.* “We have short factual reports. It didn’t go into detail about formulating the trauma or even providing a reason, understanding of that trauma and how it’s assessed and why it processed. PC has helped my practice. It’s informed me a lot, given me confidence to speak about trauma because I’m speaking from an informed standpoint. We have something that we can use and it was useful that I have a better understanding of how we target the trauma give them details of the behavior, of PC, and how you process memories.”

### Comparison across Youth and Therapist

#### Benefits

A significant amount of commonality was identified in statements and themes between youth and therapist in relation to the benefits of PC, e.g. high levels of motivation, identification of future goals, dealing with high risks, and using strategies (see Table 2). Youth, however, reported more explicit change in identity and the shift from re-experiencing to remembering. The therapist in contrast, highlighted, that youth discovered that change is possible. The therapist also identified knowledge and skill gains for herself and an increase in trauma-informed discourse in the facility.

**Table 2:** Comparison of youth gains: youth and therapist perspectives

Issue	Youth	Therapist
Impact	Emotional, behavioral, and identity change	Making good choices
Awareness	Re-experiencing to remember	Aware of gains
Motivation	Sense of purpose	Putting in a lot of effort
Future goals	Committed to achieve goals	Have and overcome difficulties
High risk	Aware of situations and consequences	Generating alternatives
Better choices	Taking responsibility and identity change	Real life change and benefit
Stress	Less distress and talking about trauma	Less distress and talking openly
Trauma memories	Sense of efficacy, past into the past	Less distress and knowing it is treatable
Anticipating future risks	Consider different outcomes and strategies	Aware, thinking, and anticipating

#### Challenges of Implementation

Most statements about the challenges of PC came from the one youth who gave the lowest ratings. In contrast to the other two youth, this youth had not addressed all his trauma memories: “Still some memories there, work to be done.” Three main ongoing needs were reported. Firstly, behavioral, “I’m still up for a bit of bother,” secondly, struggling to avoid high risk situations because of substance misuse, “When I’m sober and not on drugs,” and thirdly, problems with family relationships. It would seem then, that for one youth not all issues were addressed through one course of therapy and it may be that other programs will be necessary to address his substance misuse. Further, all three youth thought that PC may not help all young people in secure accommodation. This was based on perceptions that some youth do not see themselves as either traumatized or in need of support. Youth highlighted engagement through a good relationship with the therapist, and receiving psychoeducation (information and explanation) about the nature of exposure, the trauma response, and the process of therapy. All three highlighted the need to alert youth that PC brings back stressful memories and related negative emotions. Youth think it is important to let others know beforehand that this experience is part of putting traumatic memories and difficult emotions into the past.

Challenges identified by the therapist tended to be organizational rather youth related. This included "busy workloads caused by other work demands" and the need for "facilities to decide if they want to develop trauma-informed practice." These issues were reported as limiting access to therapy. Short duration placements because of expensive placement costs for local authorities, was reported as making it difficult to start PC where there was no guarantee of completion, “Increasingly young people are only in three weeks and you may not have time when normally the first three weeks are an assessment period.”

## Discussion

By introducing PC to a secure facility in Scotland, the current cluster case study builds on the findings of previous exploratory youth, and adult PC studies (Greenwald et al. 2015; Jarecki and Greenwald 2015; Greenwald 2014). Three youth, all with complex trauma histories, reported benefiting from PC within a trauma-informed phase model. A range of benefits were identified by youth including: a sense of accomplishment from completing therapy, increased awareness of the impact of trauma in their lives, being able to speak about trauma, and feeling that trauma was in past. Although reported retrospectively, youth moved from being traumatized, anxious, and angry to feeling less weighed under, clear headed, and proud of their achievements. Youth behavioral changes were also reported by youth and therapist. Given youth are placed in secure facilities because of risky behavior (Barron and Mitchell 2017), this is a promising finding that progressive counting (PC) may be beneficial for youth who have trauma histories, and who are motivated for treatment. Indeed, a growing number of studies support a shift away from a behavioral and criminogenic perspective to a trauma-informed approach (Ford and Blaustein 2013). Youth also reported discovering more positive identities. Such therapeutic change has the potential to lead to wide ranging posttraumatic growth and longer term gains (Calhoun and Tedeschi 2006). Such outcomes are worth including in more rigorous evaluation of PC in secure facilities.

Not all behavioral difficulties, e.g. substance misuse, were addressed and further trauma targets were still evident for one youth included in this study. Some youth, therefore, may need to revisit PC for traumas they did not address and receive other programs for non-trauma related behavior. Educational opportunities for learning, blocked by past traumatization, and community supports to nurture new attitude/skill development, may also need to be part of intervention (Ford and Blaustein 2013; Rivard et al. 2003). For youth in secure facilities, it may be that PC, as one example of a brief exposure program, is one component in a package of supports.

Gains were also reported for the therapist. The use of scripts appears to have boosted the therapist’s confidence and led to perceived skill increases in trauma-informed assessment, case formulation, and communication with others. A gain for therapist, facility, and youth was how PC, within a trauma-informed phase model, was another approach to facilitate a good quality therapeutic relationship. The therapist viewed PC as a more respectful way of working compared to a behavioral risk model, and youth valued privacy, choice, and the opportunity to be asked about and share their trauma experiences. Such relational factors have been highlighted by extensive common factor research as underpinning therapeutic effectiveness (Wampold 2015).

The current study identified a range of challenges for youth, therapist, facility, and parents in implementing PC in a secure facility. For youth, the main challenge was facing traumatic memories and coping with distressing emotions. The latter, however, was of short duration and perceived by youth to be part of the process of reduced distress overall. As with the study with therapists (Greenwald et al. 2015), youth in secure were able to tolerate PC. Further, and related to the experience of distress, youth expressed a wish to get through the trauma work more quickly, to be done with it, and enjoy life. Longer sessions also enable youth to get all the way through processing a given memory (or memories) rather than leaving one activated, but not completed. Intensive approaches to therapy then, require further exploration (Greenwald 2014). For the therapist and facility, the main challenge was to address a paradigm shift from a behavior risk model to a trauma-informed understanding. For example, ensuring there was sufficient time for therapy within a busy workload. Whole staff training in trauma-informed understandings may help to begin to address such a discrepancy (Ford and Blaustein 2013). Finally, the therapist spontaneously reported parents struggled to understand the nature of youth traumatization and how to support therapy. Again, PC embedded within a whole facility approach could include trauma-informed parent workshops and individualized parental support. The effectiveness of these developments would need a thorough assessment.

### Limitations

The only published studies to investigate PC have been conducted by its developer and the only comparison-based study examines PC against EMDR, the results of which indicate that there were no significant differences between PC and EMDR. The findings of the current cluster case study are based on unique youth and therapist experience, and although communicate perceptions of change, are not generalizable. Purposive sampling of successful cases for a beginner therapist is helpful for identifying issues for further exploration, however, findings need to be viewed with caution. The novice therapist was rated at 6–7 in terms of skill level. In contrast, more experienced and skilled therapists may achieve higher levels of impact. The sample size was small and there was no attempt to standardize assessment in these cases, rather, importance was given to participant experience. All participants were male and findings may not relate to females. The therapist was new to PC and does not represent the approach of an experienced trauma therapist. The rating of program fidelity was retrospective, albeit two months following supervision and therefore open to memory bias. Frequency counts and ranking does not necessarily infer importance of the issues. Finally, the non-inclusion of non-completing youth means the barriers to PC for such youth, have yet to be explored.

## Conclusion

This was the first study to introduce PC within a trauma-informed phase model into a secure facility in Scotland. Although PC was delivered by a novice therapist, three youth managed to complete PC and report a range of gains in line with therapy objectives. All youth were able to tolerate PC. Positively, to varying degrees, youth experienced reduced distress and a sense of trauma going into the past. Knowledge and skill were also reported for the therapist along with the development of a trauma-informed discourse in the facility. Implementation, however, was not without its challenges. One youth may need to revisit PC to deal with yet to be addressed trauma and youth requested longer more intensive PC sessions to help contain disturbing emotions. The facility had to balance PC with a pre-existing behavior risk model, and prioritizing therapy time, especially within short duration placements was difficult to achieve. In conclusion, there were sufficient perceived gains by youth and therapist to suggest further exploration of PC in secure facilities. The authors acknowledge PC is but one therapeutic option. There are other promising evidenced-based paradigms that might be utilized.

### Recommendations for Practice

The findings suggest therapists should continue to explore the effectiveness of PC for youth who have experienced cumulative trauma and present signs of posttraumatic stress in secure facilities. For facilities to implement PC appropriately, facilities will need to protect therapy delivery time for therapists. Therapists need to establish good relationships with youths, be skilled in PC, and follow treatment protocols. Implementation and evaluation of intensive PC delivery should be considered. Parents are likely to need information on understanding trauma and how to support therapy. In line with youth recommendations, youth need explanation of the purposes and methods of PC, including being alert to the short-term nature of discomfort that occurs as part of the healing process.

### Recommendations Future Research

Further research is necessary before recommending PC as an empirically-based therapeutic option, especially among potentially vulnerable populations such as youth in secure environments. All youth and therapist perceived gains in the current study need to be rigorously tested. Any differential response of boys and girls to PC requires examination. The utilization of intensive PC for youth in secure facilities requires robust research. The challenges of therapy for youth, therapists, facilities, and parents, and how these are overcome, needs further study. Case study results suggest the need for rigorous research including large sample randomized control trials and comparative studies with other promising approaches. Longitudinal evaluation is needed to assess maintenance of gains and longer term posttraumatic growth. Future studies need to include experienced therapists and non-completing as well as completing youth. Therapy sessions should be videoed and analyzed for program fidelity.
